# *In-situ* microscopy and 2D fluorescence spectroscopy as online methods for monitoring CHO cells during cultivation

**DOI:** 10.1186/1753-6561-5-S8-P76

**Published:** 2011-11-22

**Authors:** Sophia Bonk, Marko Sandor, Ferdinand Rüdinger, Bernd Tscheschke, Andreas Prediger, Alexander Babitzky, Dørte Solle, Sascha Beutel, Thomas Scheper

**Affiliations:** 1 Institute of Technical Chemistry, Leibniz University of Hanover, Germany

## Introduction

Typical methods for monitoring cultivation processes are offline analyses like cell counting, measurement of various substrates and products (e. g. glucose or lactate) as well as the online monitoring of several physical process parameters (temperature, pH-value or the concentration of dissolved oxygen).

To improve cell cultivations detailed information about important analytes should be available online. Therefore new monitoring methods need to be established, preferably as *in-situ* methods to minimize the risk of contamination.

Two different *in-situ* online-methods were used to monitor cultivations: *In-situ* microscopy and 2D fluorescence spectroscopy. Therefore CHO-K1 cells (provided by University of Bielefeld) were cultivated in a complex culture medium (TC 42, TeutoCell, Bielefeld, Germany) using a 2.5 L stainless steel reactor with a work volume of 2 L. A total of three cultivation runs were conducted.

## *In-situ* microscopy

The *in-situ* microscope (ISM), developed at our institute, offers the possibility to determine the cell density and to obtain further information about certain cell characteristics such as size, compactness and excentricity. In addition, it is possible to extract other information like cell clusters or microbial contaminations from the recorded images. In this way a general comprehensive overview of a cultivation can be obtained.

The *in-situ* microscope was immersed directly in the culture liquid using a 25 mm standard connection. During the cultivation process several images were obtained and subsequently evaluated by a self-developed algorithm which detects cells on the basis of grey scale values.

It was necessary to correlate the cells per picture with offline data determined by a Neubauer counting chamber. This correlation gives a regression coefficient (R^2^) of 0.988.

By means of this correlation the cells per picture were calibrated in reference to the counted cells per mL (root mean square error of calibration; RMSEC: 0.989). As a result it is possible to detect the cell density via ISM with an average standard error of 0.027 (pictures per cycle: 300) during further cultivations.

## 2D fluorescence spectroscopy

The 2D fluorescence spectra were collected using a Bioview® sensor (Delta, Denmark). The fluorescence spectroscopy was used to monitor glucose, lactate und glutamate during cultivation. At first it was necessary to build a calibration model for each compound. Therefore two process runs were observed by collecting 2D fluorescence spectra every 15 min. Approximately every 6 hours a sample was taken and analyzed offline for glucose, lactate and glutamate. The offline reference data and the corresponding spectra were used to build a chemometric model for prediction of the most interesting variables during a further cultivation run.

Utilizing multivariate analyzing software (Unscrambler X, vers. 10.1) a calibration model was built by partial least square regression (PLS1) for every variable. The number of factors necessary to built a model as well as the results for the regression coefficient (R^2^), the root mean square error of calibration and validation (RMSEC and RMSEV) for every variable are shown in Tab. 1.

**Table 1:** PLS-model results for glucose, lactate and glutamate.

Compound	factors	**R**^**2**^	RMSEC [g/L]	RMSEV [g/L]	RMSEP [g/L]
**Glucose**	3	0.967	0.381	0.468	0.524
**Lactate**	3	0.972	0.368	0.461	0.494
**Glutamate**	4	0.983	0.0036	0.0059	0.0155

The PLS-models were used to predict all three compounds in a third cultivation run. The standard errors of prediction (RMSEP) are also shown in Tab. 1. For glutamate the RMSEP is about 5 % compared to a maximum glutamate concentration of 0.27 g/L.

**Figure 1:**
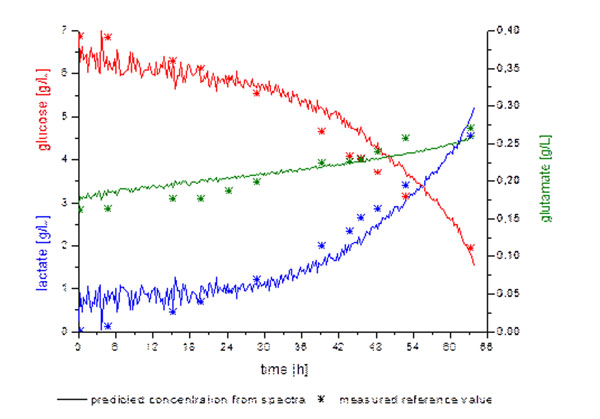
Predicted concentration gradients and their corresponding offline values.

By applying the PLS-models to the fluorescence data the concentration gradients for every single compound can be predicted (Fig. 1).

## Conclusions

In biotechnology, especially in pharmaceutical biotechnology it is important to closely monitor a cultivation process. The *in-situ* microscopy together with digital image processing and the 2D fluorescence spectroscopy in combination with chemometric tools complement one another as online monitoring methods.

It was demonstrated that the ISM can be used to monitor the cell density during a bioprocess with the same accuracy in comparison to a Neubauer counting chamber. After a successful calibration the cell density can be determined without the need for taking samples. This can reduce the risk of contamination, particularly when the cells are cultivated without the addition of antibiotics.

The Bioview® sensor could be utilized to observe three important analytes during a cultivation run with an error of prediction under 10 %. By monitoring these analytes in real time it is possible to observe sudden or unexpected changes during a cultivation and if necessary to react accordingly.

Both online methods were successfully applied to monitor the cell density as well as the glucose, lactate and glutamate concentration during cultivation.

## Outlook

Concerning the ISM further research is focused on a new prototype with precise linear stages making it possible to adjust to a certain sample volume. This should make a calibration with offline data unnecessary.

The robustness of the calibration models for the 2D fluorescence spectroscopy will be tested by adding glucose during cultivation in order to determine if the model can be applied to a fed batch cultivation.

